# Increased conditioned place preference for cocaine in high anxiety related behavior (HAB) mice is associated with an increased activation in the accumbens corridor

**DOI:** 10.3389/fnbeh.2014.00441

**Published:** 2014-12-22

**Authors:** Janine M. Prast, Aurelia Schardl, Simone B. Sartori, Nicolas Singewald, Alois Saria, Gerald Zernig

**Affiliations:** ^1^Department of General Psychiatry and Psychiatry, Experimental Psychiatry Unit, Medical University of InnsbruckInnsbruck, Austria; ^2^Department of Pharmacology and Toxicology, Institute of Pharmacy and Center for Molecular Biosciences Innsbruck, University of InnsbruckInnsbruck, Austria; ^3^Department of Psychology, University of InnsbruckInnsbruck, Austria

**Keywords:** anxiety, cocaine CPP, accumbens corridor, D1-MSNs, D2-MSNs, Egr1, c-Fos

## Abstract

Anxiety disorders and substance use disorders are strongly associated in humans. Accordingly, a widely held but controversial concept in the addiction field, the so-called “self-medication hypothesis,” posits that anxious individuals are more vulnerable for drug dependence because they use drugs of abuse to alleviate their anxiety. We tested this hypothesis under controlled experimental conditions by quantifying the conditioned place preference (CPP) to 15 mg/kg i.p. cocaine given contingently (COCAINE) in CD1 mice selectively bred for high anxiety-related behavior (HAB) vs. normal anxiety-related behavior (NAB). Cocaine was conditioned to the initially non-preferred compartment in an alternate day design (cocaine vs. saline, four pairings each). HAB and NAB mice were also tested for the effects of non-contingent (NONCONT) cocaine administration. HAB mice showed a slightly higher bias for one of the conditioning compartments during the pretest than NAB mice that became statistically significant (*p* = 0.045) only after pooling COCAINE and NONCONT groups. Cocaine CPP was higher (*p* = 0.0035) in HAB compared to NAB mice. The increased cocaine CPP was associated with an increased expression of the immediate early genes (IEGs) c-Fos and Early Growth Related Protein 1 (EGR1) in the accumbens corridor, i.e., a region stretching from the anterior commissure to the interhemispheric border and comprising the medial nucleus accumbens core and shell, the major island of Calleja and intermediate part of the lateral septum, as well as the vertical limb of the diagonal band and medial septum. The cocaine CPP-induced EGR1 expression was only observed in D1- and D2-medium spiny neurons, whereas other types of neurons or glial cells were not involved. With respect to the activation by contingent vs. non-contingent cocaine EGR1 seemed to be a more sensitive marker than c-Fos. Our findings suggest that cocaine may be more rewarding in high anxiety individuals, plausibly due to an anxiolytic effect.

## Introduction

Anxiety disorders and substance use disorders are strongly associated in humans. For example, a recent survey of 43,093 individuals in the U.S. showed that the 12-month prevalence of any drug dependence increased from 0.63 to 5.24% in individuals with a primary diagnosis of generalized anxiety disorder (GAD), which was found in 2.06% of the surveyed sample (Grant et al., 2004). GAD as the primary diagnosis increased the prevalence of alcohol dependence from 3.81 to 10.52% and of substance dependence (which can be considered the sum of drug- and alcohol dependence) from 4.07 to 13.34% (Grant et al., 2004). At first sight, these findings support a widely accepted (Khantzian, 1985; Mariani et al., 2014) but controversial (Lembke, 2012) concept in the addiction field, the so-called “self-medication hypothesis.” This hypothesis would—with respect to anxiety—posit that individuals suffering from anxiety are more vulnerable for drug abuse and dependence because they use drugs of abuse to alleviate their anxiety. However, despite all efforts at improving the study design, epidemiological surveys cannot resolve questions regarding the causal mechanisms underlying the relationship between substance use disorders and anxiety or mood disorders (Grant et al., 2004) because the temporal order of the first onset of the respective disorder (GAD vs. substance dependence) could not be ascertained beyond any doubt, allowing only the conclusion that the disorders are associated (Grant et al., 2004; Lembke, 2012) but not that one has caused the other.

Thus, the investigation of a causal relationship between anxiety and drug dependence requires the controlled conditions of an experiment in which the individual's level of anxiety can been ascertained beyond any doubt before any exposure to a drug of abuse is initiated. The high anxiety-related behavior (HAB) vs. normal anxiety-related behavior (NAB) mice, derived from CD1 mice selectively bred for their level of anxiety-related behavior displayed on an elevated plus maze (Landgraf et al., 2007), offers such an opportunity. While the HAB/NAB mouse model operationalizes several aspects of anxiety, its translational power may be greatest with respect to GAD as the HAB/NAB mouse model operationalizes trait anxiety (Sartori et al., 2011b) and as individuals suffering from GAD show high levels of trait anxiety (Rapee, 1991; Chambers et al., 2004). Therefore, one of the main goals of the present study was to investigate if cocaine, a prototypical drug of abuse that has been reported to relieve symptoms of anxiety (Khantzian, 1985), would be more rewarding for HAB than for NAB mice. We chose the conditioned place preference (CPP) paradigm (Bardo et al., 1995; Zernig et al., 2007; Prast et al., 2014) for this purpose, as CPP allows to quantify, in a drug-free state, to what degree previously neutral contextual stimuli have acquired appetitive properties after having been paired with the drug on a minimal number of occasions, i.e., only 4 times (Fritz et al., 2011; Kummer et al., 2011; Prast et al., 2014). We had previously demonstrated in rats (Prast et al., 2014) that the time spent in the cocaine associated compartment was strongly correlated with the degree of activation, i.e., expression of the immediate early gene (IEG) Early Growth Related Protein 1 (EGR1), in the whole accumbens corridor, i.e., in a region stretching from the anterior commissure to the interhemispheric border and comprising the medial nucleus accumbens core (AcbCm) and shell (AcbShm), the major island of Calleja and lateral septum (ICjM + LSI), as well as the vertical limb of the diagonal band and medial septum (VDB + MS). Human functional imaging studies have confirmed the important role of the accumbens in drug addiction (Breiter et al., 1997; Breiter and Rosen, 1999; Haber and Rauch, 2010) and anxiety (Levita et al., 2012). Therefore, the present study was designed to investigate if HAB mice would display a higher accumbens corridor activation than NAB mice (1) upon cocaine CPP conditioning and (2) after administration of non-contingent cocaine, i.e., cocaine administered not in close temporal association with any CPP conditioning procedure and (3) under baseline conditions, i.e., naive animals. We selected the CPP paradigm as we have previously focused on CPP with cocaine as a prototypical drug of abuse, intending to compare our present results with our previous findings. The CPP paradigm allows to test in a cocaine-free state, i.e., avoids the acute direct pharmacologic effect of cocaine as a confounding variable (please see Zernig et al., 2007 for a detailed discussion of these methodological issues).

Following the seminal paper by Everitt and coworkers (Lee et al., 2005), we decided to use the IEG EGR1 as a marker for neuronal activation in our paradigm (Fritz et al., 2011; El Rawas et al., 2012; Prast et al., 2012, 2014). However, the most commonly used marker for neuronal activation in the accumbens is not EGR1, but another IEG, i.e., c-Fos (Hope et al., 1992; Singewald, 2007; Muigg et al., 2009). *In vivo*, both EGR1 and c-Fos have important roles in processes such as brain development, learning, and the response to drugs of abuse or stress (Beckmann and Wilce, 1997; Perez-Cadahia et al., 2011). Of note, knockout of EGR1 in mice has shown that EGR1 expression is necessary to establish cocaine CPP (Valjent et al., 2006). In contrast, food CPP was not affected by knockout of EGR1 (Valjent et al., 2006). The final aim of the present study was to directly compare the two markers with respect to their sensitivity in our experimental paradigm, also in order to render our results directly comparable to the majority of the published studies that employ IEG expression as a marker for neuronal activation in the accumbens corridor (Prast et al., 2014).

## Materials and methods

### Subjects

Adult male HAB and NAB mice with divergent levels of trait anxiety were obtained from the breeding colony at the Department of Pharmacology and Toxicology, University of Innsbruck, Austria. The HAB and NAB mouse lines had been created at the Max Planck Institute of Psychiatry, Munich, Germany (Krömer et al., 2005) by subjecting 7 week old animals of an outbred Swiss CD1 mice population to an elevated plus maze test and by the subsequent deliberate mating of males and females with the least percentages of open arm time (i.e., marker for anxiety-related behavior), and of those with the mean percentages of open arm time, respectively. From that point on, the bidirectional inbreeding of animals with high and low anxiety-related behavior has been continued. In order to confirm the anxious phenotypes the offspring of each generation including the one used in the present studies is again tested on the elevated plus maze at the age of 7 weeks (Muigg et al., 2009) and the percentage of time in the open arms is registered. Per definition, the open arm time of HAB mice is less than 15% as compared with approximately 25–35% for NAB mice with no overlapping between the lines and with NAB mice representing the population mean of unselected CD1 mice (Sartori et al., 2011a). Animals were group-housed until the elevated plus maze test and then single-housed at around 23°C and around 55% humidity for 1–2 weeks before the start of the cocaine CPP experiment. The animals received *ad libitum* access to tap water and pellet chow, and were maintained on a 12-h light/dark cycle with lights on from 0800 to 2000 h. All animals were treated according to the ethical and scientific standards of the European Union. The present experiments were approved by the Austrian National Animal Experiment Ethics Committee.

### Place conditioning procedure

#### Housing conditions and CPP apparatus

Conditioning was conducted in a three compartment apparatus (CPP box 64 cm wide × 32 cm deep × 31 cm high) made of unplasticized polyvinylchloride. The middle (neutral) compartment (10 × 30 × 30 cm) had white walls and a white floor. Two doorways led to the two conditioning compartments (25 × 30 × 30 cm each) with walls showing either vertical or horizontal black-and-white stripes of the same overall brightness and with stainless steel floors containing either 168 holes (diameter 0.5 cm) or 56 slits (4.2 × 0.2 cm each, Kummer et al., 2011). All behavioral tests were video recorded and analyzed offline for the time spent in each compartment by an experimenter blinded to the anxious phenotype of the animals. Experiments were conducted during the light period of the cycle. Masking background noise was generated by a continuously running high efficiency particulate air (HEPA) antiallergen filter box. The CPP box was placed directly beneath a fluorescent lighting (58 W, 1 m distance).

#### Experimental groups

**Figure 1:**
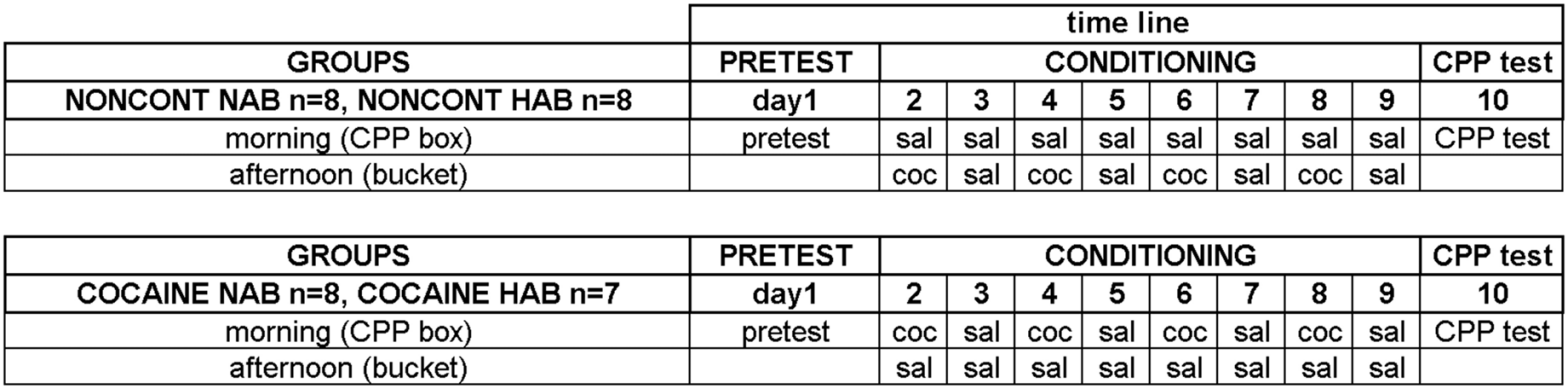
**Experimental timeline for cocaine conditioned place preference**. The detailed time line of the behavioral training is shown. See “Materials and Methods” Section for details. Experimental groups and group sizes are listed on the left; each conditioning group was treated twice per day (in the morning in the CPP box and in the afternoon in a bucket). Just briefly: the training sessions were: on day 1 the pre conditioning test (PRETEST) to assess a possible compartment bias; the CONDITIONING (day 2–9) with either cocaine (coc) or saline (sal) and the CPP test (day 10).

A detailed plan of the training schedule is shown in Figure 1. Each line was divided into three groups with comparable percentages open arm time. Animals of the NAIVE groups were sacrificed before undergoing any further treatment to investigate EGR1 and c-Fos expression in NAIVE NAB (*n* = 7) and NAIVE HAB (*n* = 8) animals. The remaining animals were divided into two groups each and were either treated with non-contingent cocaine (NONCONT NAB *n* = 8, NONCONT HAB *n* = 8) or trained for cocaine CPP (COCAINE NAB *n* = 8, COCAINE HAB *n* = 7). First, pretest bias for any of the two conditioning compartments was declared if during pretest the animal spent more time in one of the conditioning compartments in a 15 min test session. Cocaine injections were paired with the initially non-preferred side. On the following day, cocaine CPP acquisition training was started by injecting COCAINE animals intraperitoneally (i.p.) with cocaine HCl (corresponding to 15 mg/kg pure base) or saline (1 ml/kg) in an alternate day design in the morning and by putting each animal into the respective compartment inside the CPP box for 15 min immediately after the i.p. injection. The cocaine dose was chosen based on a previous review in which we extensively compared different cocaine doses (Zernig et al., 2007, p. 387) and on the CPP reviews by Bardo et al. (1995, p. 1327) who also reviewed a large number of cocaine CPP experiments. Fifteen mg/kg i.p. can be considered a high cocaine dose. In the afternoon, i.e., at least 6 h after the CPP training in the morning, the COCAINE groups received a saline injection outside of the CPP box in a clearly different context, i.e., they were injected i.p. with saline and placed for 15 min into a bedding-filled bucket (red colored polyvinylchloride bucket, diameter 20 cm, height 28 cm) before being put back into the home cage. In contrast, the animals of the NONCONT groups received i.p. saline injections before being put in either compartment of the CPP box during CPP training (i.e., they were trained for saline vs. saline) and, in the afternoon, non-contingently (i.e., not in close temporal association with any CPP conditioning procedure) received the same number of saline or cocaine injections in the same alternate day design as the COCAINE groups. This procedure assured that the NONCONT groups could not associate any compartment of the CPP box with the interoceptive effects of cocaine, thus controlling for the pharmacologic effect of cocaine as well as the handling and i.p. injection effects.

### Cocaine CPP test

On experimental day 10, the CPP test was performed 24 h after the last conditioning trial by placing the mouse in the middle (neutral) compartment of the CPP apparatus and allowing it to freely move between the three compartments for 15 min. The preference for cocaine was then calculated as time in the cocaine compartment minus time in the saline compartment (given in seconds). The mouse had to move in and out of the conditioning compartments at least five times during the CPP test for the data to be used for further analysis (which always was the case).

### Immunohistochemistry

One hour after the start of cocaine CPP test, i.e., at a time when a substantial increase in both EGR1 and c-Fos protein expression is expected (Chaudhuri, 1997; Zangenehpour and Chaudhuri, 2002), animals were deeply anesthetized with isoflurane. Animals were intracardially perfused with 0.1 M phosphate buffered saline (PBS) followed by 4% (w/v) paraformaldehyde (PFA) dissolved in PBS (pH 7.4). Brains were removed and postfixed in 4% PFA for 8 h, cryoprotected in PBS containing 10% sucrose (w/v) for 1 day and in 30% sucrose PBS at 8°C until the brains sank to the bottom. Brains were shock-frozen in isopentane (at −35°C to −39°C) and stored at −80°C until sectioning. All serial brain sections (30 μm) were cut using a Cryostat (Leica). Sections were stored in PBS containing 0.1% sodium azide at 8°C until processing for immunolabeling. A total of six randomly chosen free-floating sections from a defined anteroposterior range with respect to bregma (AP +1.34 mm from bregma to AP +0.98 mm from bregma), according to the stereotaxic mouse Atlas (Franklin and Paxinos, 2007) were processed for either EGR1 (rabbit polyclonal, 1:3000, Santa Cruz Biotechnology, sc-189) or c-Fos (rabbit polyclonal, 1:1000, Santa Cruz, Biotechnology, sc-52) immunohistochemistry. Additionally we processed three slices each for double staining of EGR1 with dynorphin (goat polyclonal anti-DYN; 1:50, Santa Cruz Biotechnology, sc-46313) to presumably label dopamine D1 receptor expressing medium spiny neurons (D1-MSNs) or the dopamine D2 receptor expressing neurons (mouse polyclonal anti-DRD2, 1:50, Santa Cruz Biotechnology, sc-5303) to label D2-MSNs, choline acetyltransferase (goat polyclonal anti-ChAT, 1:166, Millipore, AB144p) to label cholinergic interneurons; or parvalbumin (goat polyclonal anti-PV, 1:1000, Swant, PVG-214), calretinin (goat polyclonal anti-CR, 1:1000, Swant, CG1) and neuropeptide Y (goat polyclonal anti-NPY, 1:500, Novus, NBP1-46535) to label GABAergic interneurons. Another three slices each was used for doublelabeling of EGR1 with the neuron-specific nuclear protein NeuN (Mullen et al., 1991), mouse monoclonal anti-NeuN, 1:200, Millipore, MAB377) or markers for glial cells, i.e. astrocytes (Wang et al., 2013), mouse polyclonal anti glial fibrillary acidic protein, anti-GFAP; 1:200, Santa Cruz Biotechnology, sc-33673); oligodendrocytes (Najm et al., 2013), myelin basic protein (anti-MBP; 1:200, Santa Cruz Biotechnology, sc-71546) or simple tomato lectin staining (6 μg/μl, 24 h incubation, Vector laboratories, DL-1177) for microglia (Joseph and Venero, 2013). All sections were washed 3 times for 5 min each in TBS-T and only sections used for double immunohistochemistry were then incubated at 95°C for 4 min in a 10 mM citrate buffer (pH 6.0) for antigen retrieval. After a TBS-T wash, slices were incubated for 30 min in TBS containing 50 mM glycine, followed by another wash in TBS-T (3 × 5 min) and a 1 h incubation in TBS-T containing 2% BSA and 10% normal serum (normal donkey serum, Millipore, S30; or normal goat serum, Vector Labs, S-1000) depending on the secondary antibodies used. Sections were then incubated for 48 h at 8°C in 50 mM Tris-buffered saline (TBS; pH 7.4) containing 0.1% Triton-X-100 (5TBS-T) and 2% BSA with a single primary antibody against EGR1 or c-Fos or in case of the double immunolabeling with the primary antibody for EGR1 and another primary antibody for one of the markers. Sections were washed in 50 mM TBS-T for 1 h and then incubated for 2 h in 50 mM TBS-T containing 2% BSA and the donkey anti-rabbit Alexa Fluor 488-conjugated secondary antibody (1:400, Invitrogen, A21441) for EGR1 and c-Fos single labeling. For double immunolabeling we used the donkey anti-rabbit Alexa Fluor 488-conjugated secondary antibody (1:400, Invitrogen, A21441) for EGR1 together with either the donkey-anti goat Alexa Fluor 555 conjugated secondary antibody (1:400, Invitrogen, A21432) or the goat anti-mouse Alexa Fluor 555 conjugated secondary antibody (1:400, Invitrogen, A31570), depending on the primary antibody used. Finally slices were incubated 4 min with Hoechst 33258 for nuclei staining followed by an additional wash in 50 mM TBS for 1 h. Sections were then mounted onto gelatine-coated slides and coverslipped using Vectashield (Vector Laboratories, H-1000).

### Image analysis

For each immunohistochemical marker, we took representative images with a laser scanning confocal microscope (Zeiss LSM 510 Meta) at a magnification of 100×. A representative image of the EGR1 and c-Fos expression in the accumbens corridor was made at a magnification of 20×. For the quantitative analysis, we used another fluorescence microscope interfaced to a computer (Zeiss Axioplan 2 Imaging). The Pictures for the quantification were also made at a magnification of 20× in the areas of interest. Immunohistochemistry images were processed using Fiji software (fiji.sc/Fiji). The researcher who did the counting was blind to the different treatments and the counting of the positive nuclei in the unprocessed (i.e., raw) images was conducted offline using the Fiji cell counter plugin. Immunoreactivity is given as immunopositive cells per mm^2^. Only nuclei in focus of one focal plane and positive for Hoechst 33258 were counted.

### Definition of the accumbens corridor and the counting areas

**Figure 2:**
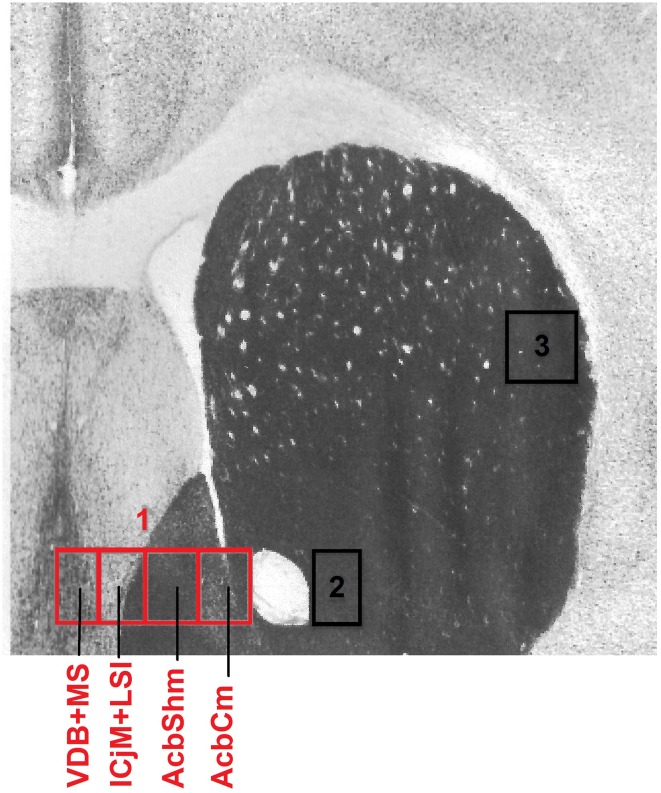
**Mouse accumbens corridor counting areas**. The investigated counting areas are displayed as individual 190 μm counting bins overlaid on an acetylcholinesterase staining at an AP location of +1.10 mm from bregma (modified from Franklin and Paxinos, 2007). Abbreviations (see Materials and Methods) follow the nomenclature of the atlas of Paxinos and Watson (2007). The numbers in the image refer to the following regions: 1, accumbens corridor; 2, AcbCl; 3, CPu.

To precisely define the borders of the regions in the accumbens corridor (Prast et al., 2014) at different anteroposterior positions with respect to bregma, we used a stereotaxic atlas by Paxinos and coworkers (Franklin and Paxinos, 2007) in which the core and shell subregions of the accumbens are distinguished by their differential acetylcholinesterase staining and histoarchitectonics (as revealed by cresyl violet staining). Abbreviations follow these authors' convention (Franklin and Paxinos, 2007) except for the “m” (for “medial”) and “l” (for “lateral”) extensions that we added to their terms “AcbSh” or “AcbC” to designate the location of these Acb subregions relative to the anterior commissure. Because we used slices only from the anteroposterior (AP) positions +1.34 mm to +0.98 mm relative to bregma, we measured the width (i.e., mediolateral extension) of the AcbCm and the AcbShm at the height of the anterior commissure at four different AP positions, i.e., at +1.34 mm, +1.18 mm, +1.10 and +0.98 mm, obtaining a mean width of 190 μm for each of these Acb subregions. Accordingly, we divided the whole accumbens corridor into 190 μm bins (Figure 2) and were able to distinguish the following regions (from medial to lateral): the nucleus of the vertical limb of the diagonal band and the medial septal nucleus (VDB + MS), the major island of Calleja and the intermediate part of the lateral septal nucleus (ICjM + LSI), the medial accumbens shell (AcbShm) and the medial accumbens core (AcbCm; Prast et al., 2012, 2014). A 190 μm strip immediately lateral of the anterior commissure represented the accumbens core lateral (AcbCl). The dorsal caudate putamen (CPu), i.e., a rectangle forming a segment with the dorsalmost curvature of the corpus callosum was also used for counting.

### Statistical analysis

Statistical differences in preference scores were calculated using a two-tailed unpaired heteroskedastic *t*-test. Immunohistochemical data (i.e., EGR1- or c-Fos immunoreactive nuclei per mm^2^) are presented as the group mean ± standard error of the mean (SEM) of individual animals (NAIVE NAB *n* = 7, NAIVE HAB *n* = 8; NONCONT NAB *n* = 8, NONCONT HAB *n* = 8; COCAINE NAB *n* = 8, COCAINE HAB *n* = 7). For each individual animal, the counts for all three slices per immunohistochemical staining were averaged before being further processed as one value per animal. Differences in EGR1 or c-Fos expression between the three groups NAIVE NAB, COCAINE NAB, NONCONT NAB or between NAIVE HAB, COCAINE HAB, NONCONT HAB animals were compared by One-Way analysis of variance (ANOVA). When the overall comparison of the groups yielded statistical significance (*p* < 0.05), subsequent comparisons between pairs of groups were performed using the least significant difference (LSD) method (Levin et al., 1994). Differences between NAB and HAB mice in the COCAINE group were calculated using a one-tailed unpaired heteroskedastic *t*-test for each brain region compared, as we expected that HAB animals show an increased EGR1 or c-Fos expression compared to NAB animals. To correlate EGR1- or c-Fos expression with the time in the cocaine compartment we calculated the Spearman's rank correlation coefficients and the one-tailed *p*-values. Analysis was performed using GraphPad Prism (www.graphpad.com).

## Results

### Cocaine CPP is increased in HAB mice

**Figure 3:**
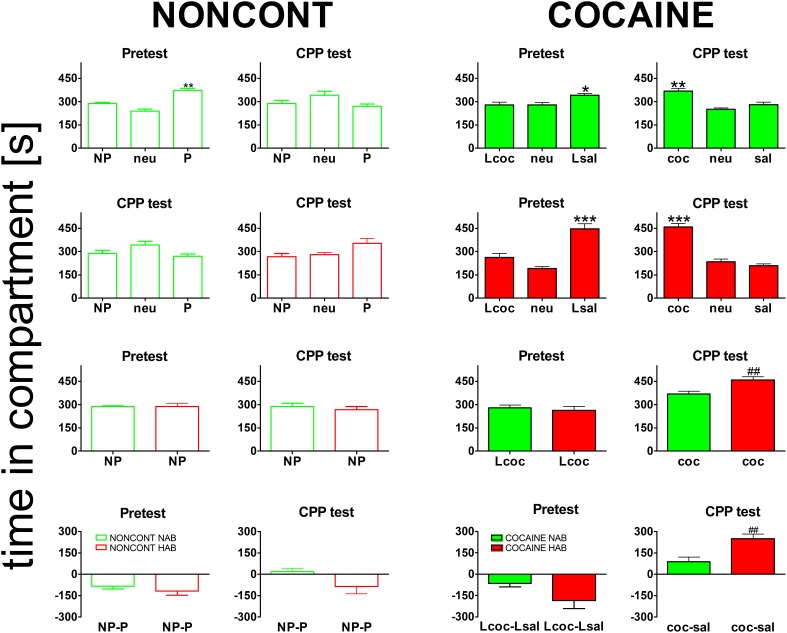
**Cocaine CPP preference is increased in high anxiety-related behavior mice**. Shown are times spent in the different compartments of the CPP box. For the NONCONT groups time spent in the initially non-preferred (NP), neutral (neu) or initially preferred compartment are displayed. For the COCAINE group time spent in the compartment later associated with cocaine- (Lcoc), the neutral (neu), or the later saline- (Lsal) associated compartment are shown for the pretest and time spent in the cocaine- (coc), neutral (neu), or saline- (sal) associated compartment are displayed for the CPP test. Time in the compartment is presented in seconds as the group mean ± SEM. Total session duration was 900 s. Data are shown for CD1 mice bred for normal- (NAB, top panel) or high anxiety-related behavior (HAB, second panel from the top) and the comparison of NAB vs. HAB animals (third panel) for both treatment groups, i.e., NONCONT (unfilled red and green bars) and COCAINE (filled red and green bars) for the pretest and the CPP test for the non-preferred compartment, which was later associated with cocaine in the COCAINE group (reexposure in a cocaine-free state). The difference between the time spent in the conditioning compartments is also shown for NAB and HAB mice as time spent in the initially non-preferred minus initially preferred (NP-P) compartment for the NONCONT groups and as time spent in the later cocaine minus later saline- (Lcoc-Lsal) associated or cocaine minus saline- (coc-sal) associated compartment for the COCAINE groups (Figure 3, bottom panel). Significant differences between the time spent in the coc and sal compartment are shown as asterisks: ^*^p < 0.05; ^**^p < 0.01; ^***^p < 0.001 (1-Way ANOVA). Statistical differences between the COCAINE NAB and COCAINE HAB group (unpaired 2-tailed *t*-test) are shown as rhombs (##*p* < 0.01).

All animals established a preference for one of the two conditioning compartments of the CPP box at the pretest day (Figure 3), i.e., before place conditioning was initiated. Interestingly, HAB mice showed a slightly higher bias for one of the compartments than NAB mice (Figure 3, bottom row). The difference was non-significant for either the NONCONT group (2-tailed *t*-test, *p* = 0.38) or the contingent COCAINE group (*p* = 0.085) alone but became statistically significant (*p* = 0.045) after pooling both groups. Time spent in the neutral compartment was excluded from further analysis as the time in the neutral compartment was not significantly different between the groups. Animals assigned to the cocaine group were conditioned with cocaine to the non-preferred compartment. At the CPP test day NONCONT NAB (Figure 3, top row, green unfilled bars) and NONCONT HAB mice (Figure 3, middle row, red unfilled bars) did not establish a preference for any compartment (1-Way ANOVA; NAB, *p* = 0.55 and HAB, *p* = 0.07). In contrast, COCAINE NAB (Figure 3, top row, CPP test, green filled bars) and COCAINE HAB animals (Figure 3, middle row, CPP test, red filled bars) developed a cocaine CPP for the cocaine associated compartment at the CPP test day (1-Way ANOVA; NAB, *p* < 0.0001 and HAB, *p* < 0.0001). COCAINE HAB animals showed an increased cocaine CPP preference compared with COCAINE NAB animals (2-sided *t*-test; *p* < 0.001, Figure 3, bottom row, CPP test).

### Cocaine CPP-induced EGR1 expression is restricted to D1- and D2-medium spiny neurons

**Figure 4:**
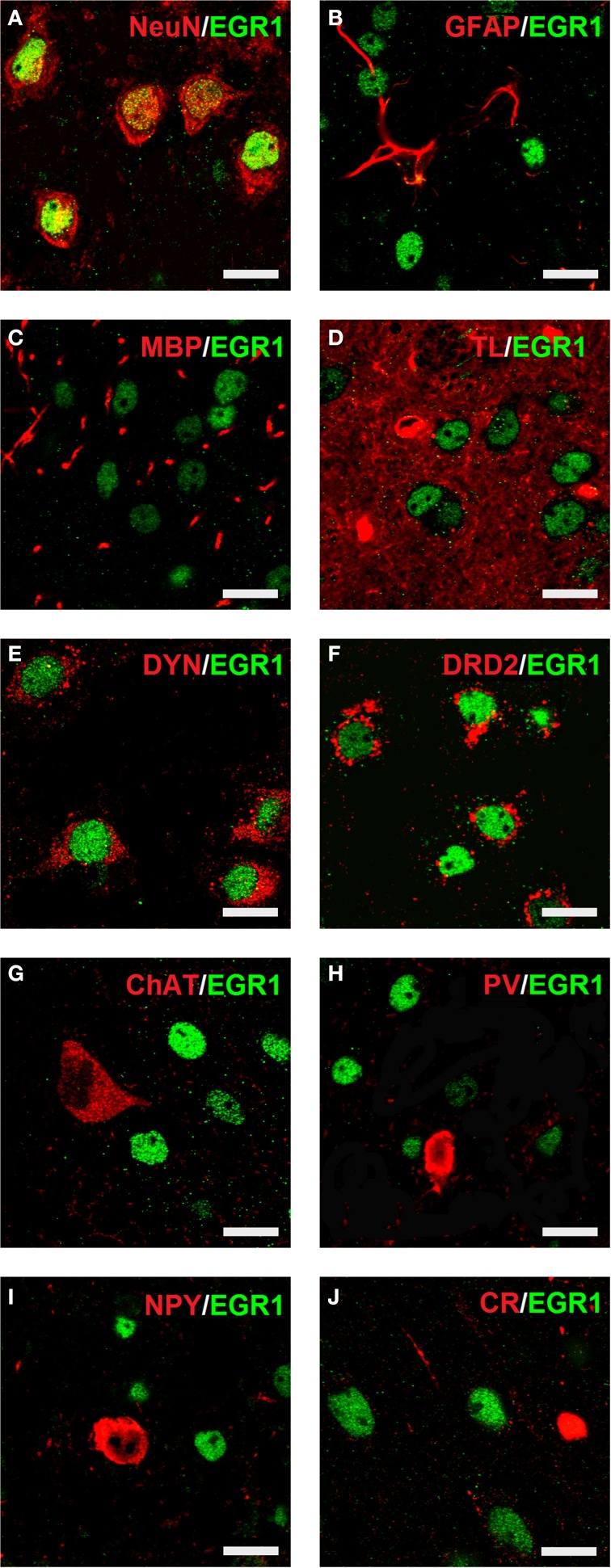
**Colocalization of neuronal markers with EGR1 expression 1 h after cocaine CPP is restricted to D1- and D2-medium spiny neurons**. Brains were harvested for double fluorescence immunohistochemistry 1 h after the start of a 15-min cocaine CPP test session. EGR1 immunoreactivity (shown in green) remained restricted to nuclei. All other neuronal markers are shown in red. Colocalization of immunoreactivity is found only in cells positive for the neuronal nuclear protein NeuN **(A)** In contrast, no colocalization with EGR1 was observed in glial cells. The employed glial markers were glial fibrillary acidic protein for astrocytes (GFAP, **B**), myelin basic protein for oligodendrocytes (MBP, **C**), and tomato lectin for microglia (TL, **D**). Colocalization of immunoreactivity was found only in neurons immunoreactive against an anti-dynorphin antibody (DYN, **E**), i.e., most likely dopamine D1 receptor expressing medium spiny neurons (D1-MSNs) or in neurons immunoreactive against an anti-dopamine D2 receptor antibody (DRD2, D2-MSNs, **F**). No colocalization with EGR1 was observed in cholinergic interneurons (marker: choline acetyltransferase ChAT, **G**) or GABAergic interneurons positive for parvalbumin (PV, **H**), neuropeptide Y (NPY, **I**) or calretinin (CR, **J**) Images were taken with a laser scanning confocal microscope with a magnification of 100× (bar size, 10 μm).

Cocaine CPP-induced an increase in EGR1 expression that remained restricted to neurons (Figure 4), as identified by colocalisation of EGR1 with the neuronal marker NeuN (panel A). A lack of colocalisation with EGR1 was observed in glial cells, positive for GFAP (panel B), MBP (panel C) or tomato lectin (panel D) in any of the treatment groups. Colocalization of EGR1 was restricted to neurons positive for DYN, most likely dopamine D1 receptor expressing neurons (D1-MSNs, panel E) and D2-MSNs positive for DRD2 (panel F). Also there was no colocalization of EGR1 with markers for cholinergic interneurons (ChAT, panel G) or GABAergic interneurons positive for PV (panel H), NPY (panel I); or CR (panel J) in any of the treatment groups. This suggested that these neuron types were not involved in mediating cocaine CPP-induced EGR1 expression and were not affected by high anxiety-related behavior.

### HAB mice show increased cocaine CPP-induced EGR1 and c-Fos expression in the accumbens corridor

**Figure 5:**
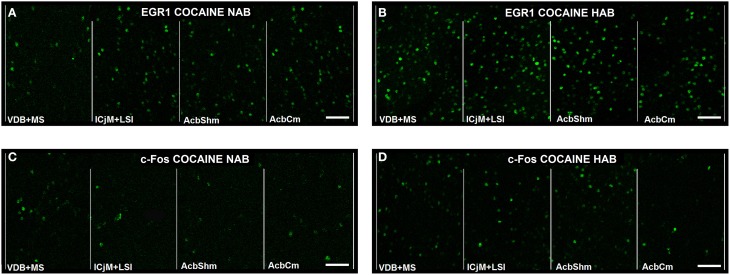
**Cocaine CPP-induced EGR1- and c-Fos expression are increased in the accumbens corridor**. Shown for each (**A–D**) are representative images of the individual 190 μm counting bins of the different accumbens corridor regions comprising, from medial to the lateral, VDB + MS, ICjM + LSI, AcbShm, and AcbCm. For abbreviations see Materials and Methods. In **(A)** Cocaine CPP-induced EGR1 expression is shown for NAB **(A)** vs. HAB **(B)** mice. Cocaine CPP-induced c-Fos expression is shown for NAB **(C)** vs. HAB **(D)** mice. Images were taken with a laser scanning confocal microscope with a magnification of 20× (bar size, 50 μm).

In order to quantify the cocaine CPP-induced neuronal activation in the accumbens corridor we assessed EGR1 and c-Fos expression by immunohistochemistry 1 h after the cocaine CPP test. Figure 5 gives an illustrative example of the cocaine CPP-induced EGR1 (Figures 5A,B) and c-Fos (Figures 5C,D) accumbens corridor activation.

**Figure 6:**
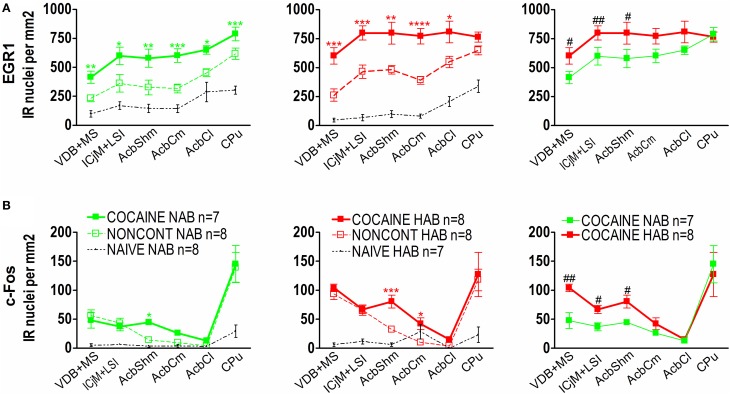
**Cocaine CPP-induced EGR1 and c-Fos expression is increased in the accumbens corridor of HAB mice. (A)** shows group means ± SEMs of EGR1-positive nuclei per mm^2^, whereas **(B)** shows group means ± SEM of c-Fos-positive nuclei per mm^2^. On the x-axis the individual regions of the accumbens corridor, the AcbCl and the CPu are displayed. The accumbens corridor comprises the VDB + MS, ICjM + LSI, AcbShm, and AcbCm (for abbreviations see Materials and Methods). The graphs in the left panel show NAB animals trained for cocaine CPP (COCAINE NAB *n* = 7, green filled squares), treated with non-contingent cocaine (NONCONT NAB *n* = 8, green unfilled squares) and NAIVE NAB animals (*n* = 8, black small dots). The graphs in the middle panel display HAB animals trained for cocaine CPP (COCAINE HAB *n* = 8, red filled squares), treated with non-contingent cocaine (NONCONT HAB *n* = 8, red unfiled squares), and NAIVE HAB animals (*n* = 8, black small dots). For reasons of clarity, significant differences (indicated by an asterisks) between treatment groups are only displayed for the comparison of animals conditioned for cocaine CPP and animals that received cocaine non-contingently (^*^p < 0.05; ^**^p < 0.01; ^***^p < 0.001, and ^****^p < 0.0001). Comparison between the NAB COCAINE and HAB COCAINE group is shown in the right panel of the figure. Statistical differences assessed by a one-tailed *t*-test between these two groups for each region of the accumbens corridor is indicated by the number sign (#*p* < 0.05; ##*p* < 0.01). The complete statistical analysis and the individual *p-*values are shown in Table 1.

Quantification showed that 1 h after the CPP test cocaine CPP-induced EGR1 expression was significantly increased in NAB (Figure 6A, left panel) and HAB animals (Figure 6A, middle panel) trained for cocaine CPP (COCAINE NAB and COCAINE HAB) compared to non-contingent cocaine treated animals (NONCONT NAB and NONCONT HAB) in the accumbens corridor (Figure 6) and in regions lateral from the corridor, i.e. the AcbCl and the CPu in the COCAINE NAB group or only in the AcbCl in the COCAINE HAB group. A comparison of COCAINE NAB and COCAINE HAB animals (Figure 6A, right panel) showed that there was a significant increase in EGR1 expression in the cocaine HAB group in the accumbens corridor, but not in lateral regions (AcbCl and the CPu).

In contrast we found that c-Fos expression in animals trained for cocaine CPP (COCAINE NAB and COCAINE HAB) was only significantly different from animals treated non-contingently with cocaine (NONCONT NAB and NONCONT HAB) in the AcbShm (Figure 6B, left and middle panel), but not in other regions of the accumbens corridor. Similarly to the increase in cocaine CPP-induced EGR1 expression in COCAINE HAB mice compared to COCAINE NAB mice we found that c-Fos expression was significantly increased in the accumbens corridor of the COCAINE HAB mice, but not in lateral regions (Figure 6B, right panel; see Table 1 for *p*-values).

**Table 1:** **Cocaine CPP-induced increase in EGR1 and c-Fos expression**.

**Immediate early gene**	**Treatment group comparison**	**Accumbens corridor**				**Lateral regions**	
		**VDB + MS**	**ICjM + LSI**	**AcbShm**	**AcbCm**	**AcbCl**	**CPu**
EGR1	COCAINE NAB vs. HAB	0.045	0.0022	0.048	0.09	0.13	0.22
EGR1	NONCONT						
	NAB vs. HAB	0.27	0.16	0.0075	0.085	0.0084	0.23
EGR1	NAIVE						
	NAB vs. HAB	0.11	0.011	0.15	0.1	0.26	0.06
EGR1	COCAINE vs. NONCONT						
	NAB	0.0031	0.0174	0.0081	0.0002	0.0287	0.0198
	HAB	0.0002	0.0002	0.0017	<0.0001	0.0112	0.052
EGR1	COCAINE vs. NAIVE NAB	<0.0001	<0.0001	<0.0001	<0.0001	0.0003	<0.0001
	HAB	<0.0001	<0.0001	<0.0001	<0.0001	<0.0001	<0.0001
EGR1	NAIVE vs. NONCONT						
	NAB	0.0177	0.0419	0.0412	0.0085	0.056	0.0001
	HAB	0.0132	<0.0001	0.0006	0.0002	0.0038	0.0002
c-Fos	COCAINE NAB vs. HAB	0.0055	0.034	0.013	0.082	0.097	0.16
c-Fos	NONCONT						
	NAB vs. HAB	0.039	0.204	0.16	0.46	0.18	0.44
c-Fos	NAIVE						
	NAB vs. HAB	0.099	0.29	0.29	0.029	0.048	0.38
c-Fos	COCAINE vs. NONCONT						
	NAB	0.99	0.56	0.0155	0.16	0.47	0.53
	HAB	0.11	0.15	0.0002	0.0245	0.14	0.097
c-Fos	COCAINE vs. NAIVE						
	NAB	0.088	0.0007	0.0005	0.0063	0.75	0.20
	HAB	<0.0001	0.0002	<0.0001	0.18	0.0029	0.011
c-Fos	NAIVE vs. NONCONT						
	NAB	0.076	0.0023	0.14	0.12	0.28	0.50
	HAB	<0.0001	0.007	0.20	0.35	0.40	0.28

*EGR1 and c-Fos expression was compared by One-Way ANOVA followed by Fisher's Least Significant Difference (LSD) test (Levin et al., 1994) between the following treatment groups for NAIVE NAB (n = 8), NONCONT NAB (n = 8), COCAINE NAB (n = 7) or NAIVE HAB (n = 7), COCAINE HAB (n = 8), and NONCONT HAB (n = 8) animals in the following brain regions: the accumbens corridor (VDB + MS, ICjM + LSI, AcbShm, and AcbCm), the AcbCl and the CPu. COCAINE NAB vs. COCAINE HAB and NONCONT NAB vs. NONCONT HAB groups were compared using an unpaired one-tailed t-test. The respective p-values for each comparison are shown in the Table. For abbreviations see Materials and Methods*.

### EGR1 and c-Fos expression correlate with the time spent in the cocaine compartment in the individual accumbens corridor regions

**Figure 7:**
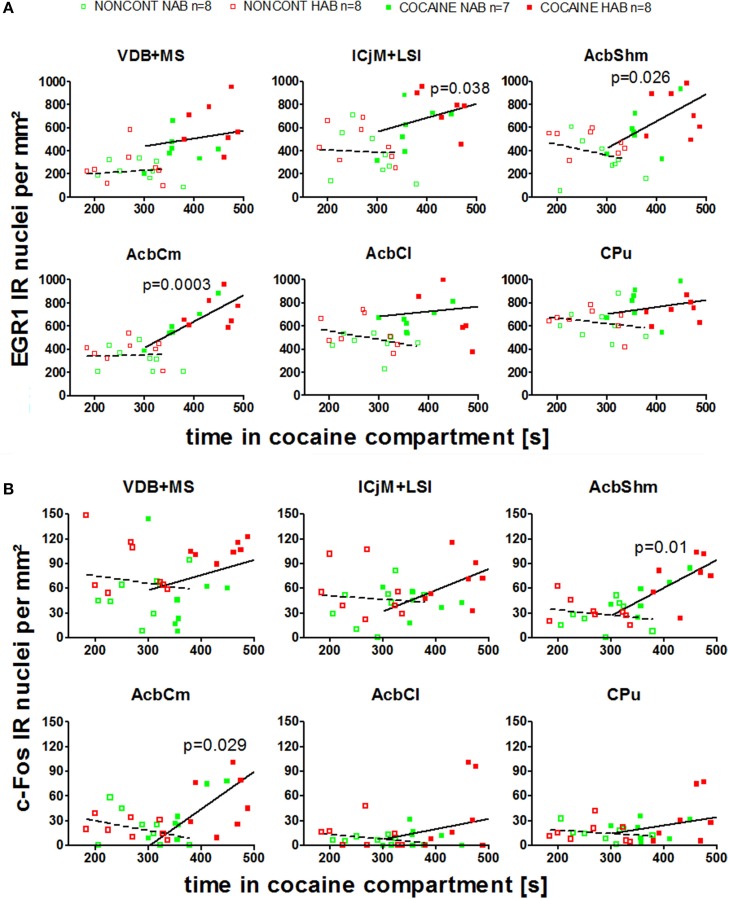
**Correlation of EGR1 and c-Fos expression in the accumbens corridor regions 1 h after the cocaine CPP test with the time spent in the cocaine associated compartment**. The correlation of EGR1 expression **(A)** or c-Fos expression **(B)** per mm^2^ vs. the time spent in the cocaine compartment during the cocaine CPP test is shown by pooling data from NAB and HAB animals. The correlation is given for each region of the accumbens corridor, the AcbCl and the CPu for animals that had undergone cocaine CPP (COCAINE NAB *n* = 7, filled green squares, filled; COCAINE HAB *n* = 8, filled red squares) as a continuous line and animals treated with non-contingent cocaine shown as dashed line (NONCONT NAB *n* = 8, unfilled green squares; NONCONT HAB *n* = 8, unfilled red squares).

We investigated if there was a correlation between the animals' preference for the cocaine-associated contextual cues (as quantified in the CPP paradigm) and neuronal activation in the accumbens corridor regions (as determined by EGR1 and c-Fos activation). As we wanted to test if this was a general phenomenon (i.e., independent of variations in conditioning), we pooled the data from the two conditioning groups, following field precedence (Golden et al., 2013; Prast et al., 2014). The respective correlational statistics for the individual treatment groups are given at the end of this paragraph. There was a correlation between the preference for the cocaine associated compartment and the EGR1 expression in the animals trained for cocaine CPP (NAB and HAB COCAINE group) in the ICjM + LSI and the AcbShm (Figure 7, panel A). In contrast there was no positive correlation for animals treated non-contingently with cocaine (NONCONT) in any of these brain areas.

Also c-Fos expression correlated with the time spent in the cocaine associated compartment in the COCAINE group in the AcbShm and the AcbCm. The NONCONT group did not show a positive correlation between the c-Fos expression per mm^2^ and the time spent in the cocaine compartment.

Spearman's rank correlation coefficients and p values for EGR1 and c-Fos expression for the different treatment groups are shown in detail in Table 2.

**Table 2:** **Correlation of cocaine CPP-induced EGR1 and c-Fos expression with time spent in the cocaine compartment**.

**Treatment group**	**Accumbens corridor**				**AcbCl**	**CPu**
	**VDB + MS**	**ICjM + LSI**	**AcbShm**	**AcbCm**		
**EGR1**						
COCAINE NAB + COCAINE HAB (pooled)	*r* = 0.30, *p* = 0.13	*r* = 0.46, *p* = 0.038	*r* = 0.50, *p* = 0.026	*r* = 0.77, *p* = 0.0003	*r* = 0.13, *p* = 0.32	*r* = 0.18, *p* = 0.25
COCAINE NAB + COCAINE HAB (pooled)	*r* = 0.20, *p* = 0.23	*r* = 0.032, *r* = 0.45,	*r* = −0.24, *p* = 0.20	*r* = −0.02, *p* = 0.47	*r* = 0.16, *p* = 0.28	*r* = 0.17, *p* = 0.28
COCAINE NAB	*r* = 0.29, *p* = 0.28	*r* = 0.54, *r* = 0.12,	*r* = 0.32, *p* = 0.25	*r* = 0.86, *p* = 0.012	*r* = 0.32, *p* = 0.25	*r* = 0.36, *p* = 0.22
COCAINE HAB	*r* = −0.07, *p* = 0.44	*r* = −0.17, *r* = 0.35,	*r* = 0.24, *p* = 0.29	*r* = 0.33, *p* = 0.21	*r* = −0.62, *p* = 0.058	*r* = 0.50, *p* = 0.11
NONCONT NAB	*r* = −0.26, *p* = 0.27	*r* = −0.40, *r* = 0.16,	*r* = −0.24, *p* = 0.29	*r* = −0.48, *p* = 0.12	*r* = −0.048, *p* = 0.47	*r* = −0.095, *p* = 0.42
NONCONT HAB	*r* = −0.36, *p* = 0.19	*r* = −0.26, *r* = 0.27,	*r* = −0.048, *p* = 0.47	*r* = −0.43, *p* = 0.15	*r* = −0.19, *p* = 0.33	*r* = −0.19, *p* = 0.33
**c−Fos**						
COCAINE NAB + COCAINE HAB (pooled)	*r* = 0.40, *p* = 0.07	*r* = 0.42, *r* = 0.059,	*r* = −0.73, *p* = 0.001	*r* = 0.68, *p* = 0.0029	*r* = 0.13, *p* = 0.32	*r* = 0.22, *p* = 0.22
COCAINE NAB + COCAINE HAB (pooled)	*r* = 0.074, *p* = 0.39	*r* = 0.05, *r* = 0.42,	*r* = −0.21, *p* = 0.21	*r* = −0.44, *p* = 0.044	*r* = −0.47, *p* = 0.033	*r* = −0.21, *p* = 0.2
COCAINE NAB	*r* = 0.036, *p* = 0.48	*r* = −0.43 *r* = 0.18,	*r* = 0.64, *p* = 0.07	*r* = 0.75, *p* = 0.033	*r* = −0.19, *p* = 0.36	*r* = −0.25 *p* = 0.30
COCAINE HAB	*r* = 0.62, *p* = 0.058	*r* = 0.0, *p* = 0.52	*r* = −0.09 *p* = 0.42	*r* = −0.29 *p* = 0.25	*r* = −0.34, *p* = 0.21	*r* = 0.25, *p* = 0.27
NONCONT NAB	*r* = −0.12, *p* = 0.40	*r* = 0.43 *p* = 0.15	*r* = −0.60, *p* = 0.066	*r* = 0.46, *p* = 0.12	*r* = −0.048, *p* = 0.47	*r* = 0.048, *p* = 0.47
NONCONT HAB	*r* = 0.69, *p* = 0.035	*r* = 0.31, *p* = 0.23	*r* = −0.31 *p* = 0.23	*r* = 0.048, *p* = 0.47	*r* = −0.05 *p* = 0.47	*r* = 0.24, *p* = 0.29

*Given are Spearman's rank correlation coefficients and p-values for the correlations of the cocaine CPP-induced EGR1 or c-Fos expression [immunopositive cells per mm^2^] and the times spent in the cocaine associated compartment [s]. Shown are correlations for data pooled from the COCAINE groups (COCAINE NAB and HAB group) and NONCONT groups (NONCONT NAB and NONCONT HAB) or correlation coefficients and p-values obtained from the individual treatment groups (NONCONT NAB, NONCONT HAB, COCAINE NAB, and COCAINE HAB) and (SOCIAL) for the different accumbens corridor regions, the AcbCl, and the CPu*.

## Discussion

Our results support the so-called “self-medication hypothesis of addiction” in that mice selectively bred for high anxiety-related behavior (HAB) and tested in a CPP paradigm found 15 mg/kg i.p. cocaine more rewarding (Figure 3) than normal anxiety-related behavior mice. Our results are in accordance with a previous study from an independent group (Pelloux et al., 2009) which found that cocaine CPP was increased in rats selectively bred for high anxiety as compared to their non-anxious conspecifics. One very likely explanation for the increase in cocaine CPP in HAB mice is that cocaine relieves anxiety in these animals. In support of this assumption, Costall and coworkers have previously shown that cocaine at a dose of 1 mg/kg i.p. or more, administered twice daily for several days, increased the time spent in the bright compartment of a black and white box and increased other measures indicative of an anxiolytic effect in male albino BKW mice, whereas cocaine withdrawal produced a temporary anxiogenic effect (Costall et al., 1989). These data reflect the human situation in that cocaine user self-report to consume cocaine to relieve symptoms of anxiety (Khantzian, 1985). In contrast, Estelles and coworkers have found variable effects of cocaine on elevated plus maze behavior of male OF1 mice (Estelles et al., 2007). These effects depended on the pattern of cocaine administration, the age of the mice, and the housing conditions, without yielding a general pattern (Estelles et al., 2007).

By subjecting the animals to only four cocaine exposures, each separated by 2 days, we tried to minimize a confounding effect that jeopardizes all human epidemiological studies (Grant et al., 2004; Lembke, 2012), i.e., that drug withdrawal may cause symptoms of anxiety (see also the mouse data by Costall et al., 1989, above) the alleviation of which leads to renewed consumption of the very same drug that had been taken to alleviate non-drug-related anxiety in the first place, thus blurring a simple cause-effect relationship between anxiety and drug consumption. We also tested the effect of non-contingent (i.e., CPP training procedure independent) cocaine and found no difference between HAB and NAB mice in the CPP test (pitching two saline injections against each other during CPP training), demonstrating that there were no differences between HAB and NAB mice with respect to CPP-relevant pharmacological cocaine effects. Interestingly, while non-contingent cocaine did not differentially affect behavior in the CPP paradigm, non-contingent cocaine in HAB vs. NAB mice increased the expression of EGR1 in the AcbShm and the AcbCl, whereas c-Fos expression was increased in the VDB + MS (Figure 6 and Table 1). This suggests that HAB mice may also be more sensitive to the direct pharmacological effects of cocaine. Of note, HAB mice also showed a higher EGR1 baseline expression in the ICjM + LSI compared to NAB mice, whereas c-Fos baseline expression was increased in the AcbCm, but lower in the AcbCl compared to NAB mice. To complicate matters further, cocaine and other psychostimulants in animal experiments have been shown (for review see Zernig et al., 2007) to produce appetitive and aversive effects (plausibly including anxiogenic effects) at the very same doses of 0.03–3 mg/kg i.v. In the present study, however, the appetitive/rewarding effects of cocaine must have prevailed over possible anxiogenic effects: Otherwise, HAB mice would most likely have shown less cocaine CPP than NAB mice or would even have developed conditioned place aversion to cocaine, which was clearly not the case.

Paralleling the increased cocaine CPP (Figure 3), the cocaine CPP-induced expression of the IEGs EGR1 and c-Fos in neurons (Figure 4) was increased in many regions of the accumbens corridor of HAB vs. NAB mice (Figure 5), most notably the medialmost ones, i.e., VDB + MS, ICjM + LSI, and AcbShm (Figure 6, rightmost panels, and Table 1). In contrast, regions lateral of the accumbens corridor (AcbCl and CPu) were not differentially affected in HAB vs. NAB mice (Figure 6) despite the pronounced increase in cocaine CPP in HAB mice. These results mirror previous findings by our group in rats in that the regions lateral of the accumbens corridor, i.e., the accumbens core lateral of the anterior commissure and the dorsal striatum, are much less affected by the cocaine CPP reacquisition and its inhibition by a previous history of dyadic social interaction than the regions within the accumbens corridor (Prast et al., 2014).

This suggests that the accumbens corridor regions (Prast et al., 2014) are not only important for the acquisition/expression of drug reward as operationalized in the CPP paradigm, but also for anxiety-related behavior. Data from human imaging studies support the idea that the Acb plays a role in reward (Breiter et al., 1997; Breiter and Rosen, 1999; Haber and Rauch, 2010) as well as anxiety (Levita et al., 2012). The nucleus accumbens proper has been shown to mediate anxiety (Ahmadi et al., 2013) in animal experiments (Muigg et al., 2009; Ahmadi et al., 2013). Interestingly, in HAB mice deep brain stimulation of the lateral nucleus accumbens core causes anxiolytic effects in HAB- but not NAB mice (Schmuckermair et al., 2013). Clinically, deep brain stimulation of the accumbens region has been shown to alleviate symptoms of anxiety (Sturm et al., 2003; Bewernick et al., 2010). Of note, Schlaepfer and coworkers (Bewernick et al., 2010) found that deep brain stimulation of the accumbens in treatment-resistant depressive patients did not only increase the number of “positive activities,” but also decreased symptoms of anxiety and depression. Thus, electrical stimulation of the accumbens region in humans not only increased the frequency of motivated behavior—a function for which the accumbens is well-known for (Salamone and Correa, 2012)—but also relieved anxiety. As the amygdala is known to mediate memories with emotional content (Herry et al., 2010; Lüthi and Lüscher, 2014) and to project extensively to the AcbShm (Heimer et al., 1997), it is plausible that amygdala-AcbShm projections modulate the effect of anxiety on AcbShm MSN activation. Regions medial of the nucleus accumbens i.e., the major island of Calleja and intermediate part of the lateral septum as well as the medial septum/diagonal band complex have also been shown to be affected by rewarding/reinforcing effects of drugs of abuse (Mahler and Aston-Jones, 2012; Prast et al., 2014) or by anxiety (Menard and Dallas, 1996; Degroot and Treit, 2003; Razavi et al., 2014). For instance, it has been shown that neurons in the lateral septum were specifically activated during cue-induced cocaine seeking (Mahler and Aston-Jones, 2012) or that the cocaine CPP reacquisition-induced EGR1 expression was increased in the medial septum/diagonal band complex and also in the major islands of Calleja and the intermediate part of the lateral septum (Prast et al., 2014). A role of the medial and lateral septum in anxiety has been shown by lesioning of the lateral and medial septum, which leads to reduced anxiety behavior on the elevated plus maze (Menard and Dallas, 1996). Administration of muscimol into the medial septum also induced anxiolytic effects in a shock probe burying test (Degroot and Treit, 2003). There is a lot of evidence indicating that these regions form a functional continuum which we termed “accumbens corridor” (see discussion in Prast et al., 2014). All these findings suggest that the accumbens corridor might be important for mediating anxiety-related behavior (for the LSI, see Muigg et al., 2009) and cocaine CPP-induced EGR1- (Prast et al., 2014 and present study) and c-Fos expression (present study). With respect to baseline EGR1- and c-Fos activation of accumbens corridor D1- and D2-MSNs in high anxiety- vs. normal anxiety mice, which may have given us an indication if anxiety *per se* results in an increased activation of accumbens corridor MSNs, our findings are equivocal: HAB mice showed an increased baseline EGR1 expression in the ICjM + LSI (Figure 6A and Table 1), whereas baseline c-Fos activation was increased in a different region, i.e., the AcbCm (Figure 6B and Table 1), allowing no firm conclusion.

We found that the cocaine CPP-induced EGR1 activation in the accumbens corridor in CD1 mice bred for normal or high anxiety-related behavior is mediated by D1- and D2-medium spiny neurons (Figure 4). In contrast, we did not find a contribution of either cholinergic interneurons and GABAergic interneurons or glial cells in mediating the global EGR1 response (Figure 4). This is in accordance with previous results from our group showing that only D1- and D2-MSNs mediate the cocaine CPP reacquisition-induced EGR1 expression (Prast et al., 2014). To summarize, cocaine CPP, either upon reacquisition in normal anxiety rats (Prast et al., 2014) or upon acquisition/expression in NAB- and HAB mice (present study) activates only D1- and D2-MSNs and no other neuronal or glial population in the accumbens corridor.

A role of EGR1 in anxiety was demonstrated by Ko et al. (2005) who found that EGR1 knock out (KO) C57BL/6 mice showed a roughly 10-fold increase in open arm time in the elevated plus maze, indicating that a lack of EGR1 in the whole brain strongly reduces anxiety. The EGR1 KO mice also displayed impaired memory for late auditory cue-conditioned fear induced by *multiple* electric shocks, whereas context and auditory fear memory induced by a *single* shock and extinction of context and auditory fear memory were not affected. Finally, EGR1 KO decreased synaptic potentiation in the amygdala and cortex.

With respect to the direct comparison of the neuronal activation markers EGR1 vs. c-Fos, the effect of contingent vs. non-contingent cocaine was more pronounced for EGR1 than c-Fos in the very same brains (Figure 6). To emphasize, despite yielding a higher baseline immunohistochemical signal, EGR1 produced more pronounced increase by non-contingent cocaine than c-Fos. When comparing cocaine CPP associated activation Figure 6, rightmost panels), however, the relative increase in immunopositive nuclei was comparable between the two markers, although EGR1 yielded higher absolute values. Taken together, the present findings suggest that EGR1 may be a more sensitive marker for the direct pharmacological vs. the conditioned (“psychological”) effects of cocaine in our paradigm than c-Fos. It has been reported that the sensitivity of c-Fos induction is not uniform in all regions of the brain (Chaudhuri, 1997; Chaudhuri et al., 2000) which could explain the observed differences between c-Fos and EGR1 expression (Figure 6), with c-Fos showing a very low tonic (baseline) activation vs. EGR1 showing a high tonic activation. The finding that expression of EGR1 and c-Fos is not identical after the same stimuli has also been reported by others (Cole et al., 1989; Nguyen et al., 1992). The differences can be explained by the fact that, although EGR1 and c-Fos have a common activation pathway (Chaudhuri, 1997; Herdegen and Leah, 1998; Zhai et al., 2008), there are differences in the induction (Zangenehpour and Chaudhuri, 2002), DNA binding sequence and binding to the promoter region (Chaudhuri, 1997; Herdegen and Leah, 1998), expression (Sassone-Corsi et al., 1988; Herdegen and Leah, 1998; Ishida et al., 2000; Slattery et al., 2005) and posttranslational modification (Chaudhuri, 1997).

There was a correlation between the animal's behavior (i.e., the time spent in the cocaine associated compartment) and the degree of cocaine CPP-induced EGR1 expression, and, to a lesser degree, the cocaine CPP-induced c-Fos expression in some of the accumbens corridor regions (Figure 7), suggesting that cocaine CPP is broadly affecting the accumbens corridor. This is in line with our previous experiments (Prast et al., 2014) in which we also found a correlation between the time spent in the cocaine associated compartment and the EGR1 expression per mm^2^(for a detailed mouse vs. rat comparison with regard to our experimental paradigms see Kummer et al., 2014). Moreover, the correlation was observed in both studies regardless of the conditioning protocol, as we tested the acquisition/expression of cocaine CPP in the present study and a cocaine CPP reacquisition paradigm in our previous study (Prast et al., 2014).

Interestingly, the HAB mice not only showed a pronounced increase in cocaine CPP compared to NAB mice but also demonstrated a slightly higher but non-significant bias for one of the conditioning compartments during the pretest than NAB mice. This slightly bigger side bias became statistically significant (*p* = 0.045) only after pooling COCAINE and NONCONT groups. As we used a biased CPP procedure, i.e., conditioned cocaine to the initially non-preferred side, we cannot exclude that HAB mice may have experienced a higher level of stress and/or anxiety in the initially non-preferred side, thus adding to a likely anti-stress and/or anxiolytic effect of cocaine which may have amplified the cocaine CPP in HAB mice even further. Of note, this phenomenon supports the notion that the cocaine reward in the present study was due to its anxiolytic effect. In a similar vein, placing the CPP boxes under fluorescent light may also have produced a higher level of stress/anxiety, rendering cocaine CPP even stronger in HAB mice because of an anxiolytic effect.

In conclusion, the present findings support the self-medication hypothesis of addiction in that cocaine proved to be more rewarding in a mouse model that reflects trait anxiety. Our results also suggest an important role of the accumbens corridor in mediating the rewarding properties of cocaine based on its likely anxiolytic effect. As shown previously in Sprague Dawley rats using a different CPP procedure, the preference for cocaine (measured as the time spent in the cocaine associated compartment) was again correlated with the amount of EGR1 activation (present study). Overall, the IEG EGR1 seemed to be a more sensitive marker than c-Fos in our behavioral paradigm. One avenue of future research is to differentiate the contribution of each accumbens corridor region by targeted activation/inactivation. It would also be worthwhile to investigate if antidepressant/anxiolytic treatment or deep brain stimulation is able to inhibit the increase of cocaine CPP in HAB mice and inhibit the cocaine CPP-induced IEG expression.

## Author contributions

Janine M. Prast, Alois Saria, and Gerald Zernig designed the experiments with the support of Nicolas Singewald and Simone B. Sartori Animals were provided by Nicolas Singewald and Simone B. Sartori, Janine M. Prast, and Aurelia Schardl performed the experiments. Janine M. Prast, Gerald Zernig, Aurelia Schardl and Alois Saria analyzed the data. Janine M. Prast and Gerald Zernig wrote the paper.

### Conflict of interest statement

The authors declare that the research was conducted in the absence of any commercial or financial relationships that could be construed as a potential conflict of interest.
